# The impact of rheumatoid arthritis on foot function in the early stages of disease: a clinical case series

**DOI:** 10.1186/1471-2474-7-102

**Published:** 2006-12-21

**Authors:** Deborah E Turner, Philip S Helliwell, Paul Emery, James Woodburn

**Affiliations:** 1 Department of Podiatry, University of Huddersfield, Huddersfield, UK; 2 Academic Unit of Musculoskeletal Disease, University of Leeds, Leeds, UK; 3 HealthQWest, School of Health & Social Care, Glasgow Caledonian University, Glasgow, UK

## Abstract

**Background:**

Foot involvement occurs early in rheumatoid arthritis but the extent to which this impacts on the structure and function leading to impairment and foot related disability is unknown. The purpose of this study was to compare clinical disease activity, impairment, disability, and foot function in normal and early rheumatoid arthritis (RA) feet using standardised clinical measures and 3D gait analysis.

**Methods:**

Twelve RA patients with disease duration ≤2 years and 12 able-bodied adults matched for age and sex underwent 3D gait analysis to measure foot function. Disease impact was measured using the Leeds Foot impact Scale (LFIS) along with standard clinical measures of disease activity, pain and foot deformity. For this small sample, the mean differences between the groups and associated confidence intervals were calculated using the *t *distribution

**Results:**

Moderate-to-high foot impairment and related disability were detected amongst the RA patients. In comparison with age- and sex-matched controls, the patients with early RA walked slower (1.05 m/s *Vs *1.30 m/s) and had a longer double-support phase (19.3% *Vs *15.8%). In terminal stance, the heel rise angle was reduced in the patients in comparison with normal (-78.9° *Vs *-85.7°). Medial arch height was lower and peak eversion in stance greater in the RA patients. The peak ankle plantarflexion power profile was lower in the patients in comparison with the controls (3.4 W/kg *Vs *4.6 W/kg). Pressure analysis indicated that the RA patients had a reduced lesser toe contact area (7.6 cm^2 ^*Vs *8.1 cm^2^), elevated peak forefoot pressure (672 kPa *Vs *553 kPa) and a larger mid-foot contact area (24.6 cm^2 ^*Vs *19.4 cm^2^).

**Conclusion:**

Analysis detected small but clinically important changes in foot function in a small cohort of RA patients with disease duration <2 years. These were accompanied by active joint disease and impairment and disability.

## Background

Small joint inflammation in the hands and feet are the hallmark of early rheumatoid arthritis (RA). Clinical studies suggest foot pain may be problematic in about one-third of patients in early disease with more frequent involvement of the metatarsophalangeal (MTP) joints (34%) in comparison with the midtarsal (4%) and ankle (20%) regions [1]. Radiographically, the joints of the feet show damage more often, and earlier than the joints of the hand [2]. Furthermore, in patients with disease duration of <12 months, edema, synovitis and erosion can be detected by magnetic resonance imaging (MRI) at the MTP joints in patients whose hands are normal [3]. Ultrasonography has also revealed MTP joint effusion, flexor tenosynovitis and plantar bursa in early disease [4]. By contrast, tarsal/ankle joint pathology occurs later in the disease with joint space narrowing and erosion in the ankle found in less than 2% of patients within the first 3 years [5,6].

Whilst imaging enables detection of joint and soft-tissue pathology, clinical signs of foot disease are often more subtle in early RA and data are lacking. In the forefoot, abnormal MTP joint alignment, subluxation and stiffness were found in 25% of patients within 3 years of disease onset [7]. In the hindfoot, <10% of cases had moderate to severe deformity by 5 years, although gait analysis can enable detection of collapsing pes valgus within 3 years [7,8]. Normal foot joint motion is necessary to allow the body to progress over the supporting foot during stance. This complex structure must facilitate weight acceptance and transfer, contribute shock absorption and stability and distribute pressure evenly on the plantar surface. The consequences of persistent synovitis in terms of disrupting these functions are well documented for established RA [8-13] but the association between joint damage in the feet, impairment and function has not been evaluated in early disease. Nevertheless, strong recommendations have been made for careful attention to foot problems in early disease with an emphasis towards correcting underlying biomechanical faults, chiefly through the use of orthotic devices [14,15].

We require a better understanding of the impact of RA in the foot and we have recently developed metrics that accurately and reproducibly measure impairment and disability [16]. Moreover, three-dimensional (3D) gait analysis permits high-definition measurement of foot function and we have successfully studied this in patients with well-established disease [8,12,13]. Therefore, the aim of this study is to compare clinical disease activity, impairment, disability, and foot function in normal and early rheumatoid arthritis (RA) feet using standardised clinical measures and 3D gait analysis.

## Methods

### Patients

Twelve patients with a history of rheumatoid arthritis ≤2 years duration (from symptom onset) were consecutively recruited from the Early Arthritis Clinic at the Leeds General Infirmary, UK. All patients had foot problems meriting referral to the rheumatology specialist podiatrist. Twelve community dwelling adults with no history of inflammatory arthritis or musculoskeletal disease involving the lower limb and foot were recruited for comparison. This study received ethical approval from Harrogate Local Research Ethics Committee (REC reference number 04/03/03).

### Clinical data

The age, sex, disease duration, disease activity score (using 28 joint counts), erosive state (Larsen score ≥2) of MTP joints, body height and mass and current disease modifying anti-rheumatic drugs (DMARD) were recorded for each patient. Using standardised techniques, a single clinician (JW) recorded swollen (SJC) and tender (TJC) joint scores in the foot at the ankle, subtalar, calcaneocuboid, talonavicular, metatarsophalangeal, interphalangeal joint of the hallux and proximal interphalangeal joints of the lesser toes (range 0–14). The number of patient reported painful (PJC) joint sites as directly indicated on the foot or on a foot map if the feet could not be reached was also recorded (range (0–14). Disease impact was measured using the Leeds Foot Impact Scale [16]. This self completed questionnaire comprises two subscales for impairment/footwear (LFIF_IF_) and activity limitation/participation restriction (LFIS_AP_). The former contains 21 items related to foot pain and joint stiffness as well as footwear related impairments and the latter contains 30 items related to activity limitation and participation restriction [16]. Fore- and rearfoot deformities were quantified using the Structural Index (SI) score [9]. This scale summates hallux valgus, 5^th ^MTP exostosis, claw/hammer toe and MTP subluxation deformities for the forefoot (range 0–12) and calcaneus valgus/varus, ankle range of motion and pes planus/cavus deformities for the rearfoot (range 0–7). The varus/valgus alignment of the heel (RSFP) was measured using a standard hand-held goniometer [13].

### Gait analysis

A six-camera 60 Hz video-based motion analysis system (Falcon System, Motion Analysis Corporation, Santa Rosa, CA, USA) was used to track the motion of 22 spherical reflective targets (10 mm diameter) placed on the shank and foot [13]. Visual3D software (C-motion, Inc., Rockville, MD, USA) was used to build segmented foot models based on the coordinates of the markers placed over specific anatomical landmarks during static calibration. Marker placement and segment models were based on those described by Carson *et al *(2001) [17]. The model comprised the shank, rearfoot, forefoot, and hallux segments. To permit calculation of ankle joint moments and power a single foot segment was also created. A single marker was also located over the tuberosity of the navicular to measure arch height during the foot flat period of stance [13]. Ground reaction forces and the distribution of pressure on the plantar surface were simultaneously measured using dual-mounted force (Bertec Corporation, Columbus, OH, USA) and pressure (EMED-ST, Novel GmbH, Munich, Germany) plates. An instrumented walkway (GAITrite, CIR systems, Clifton, NJ, USA) was used to capture spatiotemporal gait parameters.

From an average of five walking trials a pre-determined core set of foot biomechanical variables was extracted from the gait measurements [8-13]. These included walking speed and double-support as objective measures of global function [9,12,13]. Movement of the single foot segment relative to the floor was used to extract initial foot contact and the terminal stance heel rise angles in the sagittal plane (+ dorsiflexion/- plantarflexion). From the plantar pressure analysis, we derived the time as a % of stance when the centre-of-pressure reached 50% of foot length. These three measurements were used to describe the rocker function of the foot [10,11,13]. Arch height was defined as the lowest navicular height during the foot flat period of stance derived from the vertical (z) coordinate of the navicular motion tracking marker. From the movement of the rearfoot relative to the shank in the frontal plane, peak eversion motion was derived during stance (+ inversion/- eversion). These two variables provided measurements for planovalgus deformity for the mid- and rearfoot [8,12,13]. Kinetic variables describe the forces responsible for producing movement and peak vertical (+) ground reaction forces acting perpendicular to the plantar foot surface during the loading response and terminal stance were selected for analysis [10]. The dorsi/plantar flexion muscular moment and power acting about the ankle joint in the sagittal were also estimated [11]. For the recorded plantar pressure distributions, an automated software routine was used to define the forefoot (tarso-metatarsal to distal metatarsal head area) and toe (combined hallux and lesser toes) regions of the foot. We derived the peak pressure for the forefoot and the contact area for the toes regions as metrics to capture functional changes associated with MTP deformity which is highly prevalent in RA. Similarly, contact area in the mid-foot (defined as the region from the distal plantar heel to the tarso-metatarsal junction) was measured to assess collapse of the medial longitudinal arch which is associated with pes planovalgus [12].

### Statistical analyses

The clinical and biomechanical variables are summarised as mean (SD) or median (range). The small sample size did not permit formal hypothesis testing therefore to compare differences between the study groups, the mean differences and associated confidence intervals were calculated using the *t *distribution. A group ensemble average, displayed as a time series graph, was prepared for 4 selected biomechanical variables.

## Results

### Demographic and clinical characteristics

Twelve female RA patients with a median age of 46 years (range 27–63) were studied (Table 1). Able-bodied control subjects were closely matched by age and sex. Of the 12 patients studied; 3 had erosive MTP joints; 10 were treated with DMARD agents; 3 had high disease (DAS28 >5.1); 5 medium disease (DAS28 >3.2 ≤5.1); and 4 low disease (DAS28 ≤3.2).

### Foot impairments

The 3 patients with high disease activity had moderate-to-high (LFIS_IF _>7 point, LFIS_AP _>10 points) levels of foot impairment and disability on the LFIS subscales combined with a high number of swollen, tender and painful foot joints and only mild deformity (SI ≤ 3) in the forefoot (Table 1). One patient had a passively correctable varus heel deformity on weight-bearing (patient 2, 6° varus).

Moderate levels of foot impairment and related disability were observed in the 5 patients with moderate disease activity. All 5 had tender joints with most frequent involvement at the MTP joints. Three from 5 patients had self reported painful foot joints. 2/5 patients had swollen foot joints. Four from five cases had valgus heel deformity in excess of the median for the able-bodied control subjects (range 4 to 9° valgus) with three cases showing early signs of pes planovalgus on the SI score. Two patients had marked deformity in the forefoot.

In the series of 4 patients with low disease activity there was a mixed pattern of clinical disease activity, foot related impairment and disability and deformity. These patients exhibited; low levels of clinical synovitis; residual impairment characterised by low-moderate LFIS subscale scores; and deformity notably of the rearfoot with 3 cases showing planovalgus and 1 case varus foot deformity.

### Gait analysis

**Figure 1:**
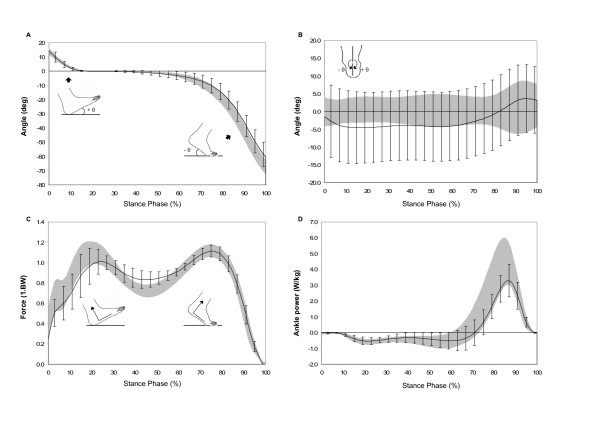
Selected gait parameters normalised for 100% stance (each graph is individually scaled and the gray band represents the mean ± 1 SD for the able-bodied adults and the solid line is the mean ± 1 SD error bars for the RA patients. A – Initial foot-to-floor and terminal stance heel rise angles during stance, measured as the angle of the plantar surface of the foot to the horizontal. Positive angles decreasing to zero indicate increasing foot-to-floor contact, zero indicates foot flat, and increasing negative angles indicate progressive heel rise; B – frontal plane motion of the rearfoot in the shank coordinate system during stance phase (+ inversion/- eversion); C – ground reaction forces normalised to body mass acting perpendicular to the plantar aspect of the foot; D – sagittal plane ankle joint power during stance).

Several differences were exhibited by the early RA patients in comparison with the able-bodied control subjects. A reduction in walking speed and an increase in double-support time indicated marked changes in global function (Table 2). The initial foot-to-floor contact angle was unchanged but during terminal stance the heel rise was reduced on average by 6.7° (Table 1, Figure 1A). Kinematics of the mid- and rearfoot showed an overall trend towards a slight reduction in medial longitudinal arch height accompanied by an exaggerated everted heel posture throughout the stance phase (Figure 1B). Both measurements were highly variable as two patients (patients 2 and 10) had varus heel deformity, thereby causing the medial longitudinal arch to rise and the rearfoot to invert.

The peak vertical ground reaction force was slightly blunted during the loading response and normal during terminal stance (Figure 1C). The peak plantarflexion net muscular moment was unchanged but there was a trend towards a reduction in the peak ankle joint power (mean difference 1.3 W/kg). The centre-of-pressure at 50% of foot length occurred at the same time (~44% of stance). Plantar pressure distribution showed a slight reduction in lesser toe contact area; an increase in midfoot contact area; and an average increase in the peak forefoot pressure of 22%.

## Discussion

There has been an increasing use of gait analysis as an objective measure of foot function in RA [8-13]. This prompted us to investigate the relationship between impairment, clinical disease activity and function in a group of RA patients with early disease. Our study showed moderate-to-high levels of disease impact in the foot associated with varying levels of clinical disease activity, pain, deformity and altered function. The LFIS is a new validated measure of disease impact in the foot developed by our own group [16]. Moderate to high levels of impairment and foot related disability were observed in these early patients. Indeed, the median scores were similar to those currently observed amongst our own clinic patients with established disease of >5 years. Therefore, LFIS like HAQ (a disability metric which predicts poor outcome when high at disease onset) may be an important predictive measure of future localised disease impact [16,18].

Despite DMARD treatment in 10/12 patients, all of the early patients were suffering foot pain which may have been related to inflammation or impairment or both. Inflammatory processes may be the predominant determinant of disability in early RA, with structural abnormalities leading to functional impairment in well established disease. In the foot, for example, synovitis is thought to reflect underlying systemic disease activity [19] whilst pain and disability correlate strongly with patients self perception of foot pain but not disease duration, joint damage or systemic drug treatment [20]. Furthermore, impairment of gait and mobility are associated with well recognised patterns of pain and foot deformity [8-13]. These problems reported in cohorts of patients with well-established RA are not very different from those reported in this study. We were able to detect functional limitation in gait in patients with <2 years disease duration characterised by slow walking speed and increased double-support and this may suggest early adaptation to underlying systemic and local disease activity and impairments [9-13].

Gait analysis has improved out understanding of foot function in established RA but has not been reported in early disease. Typical forefoot deformities such as hallux valgus, MTP joint subluxation and hammer toe deformities (component scores of the SI) were detected in 75% of the early RA patients and 50% of the control subjects. Although not staged by severity, deformity was worse in the RA cases resulting in a reduced weightbearing contact area for the toes and elevated peak pressures at the plantar MTP joints. Furthermore, plantar pressures are higher at MTP joint which are eroded and of three patients with erosive changes, two had peak values outside normal limits (>2 SD above normal mean limit) [21]. If untreated, these stresses may be associated with persistent or worsening symptoms and the development of secondary pressure lesions such as callus, bursa and ulceration [22,23]. However, some patients can off-load these painful sites by avoiding weight transfer to the forefoot. O'Connell *et al *(1998) reported disruption to the rocker function of the foot characterised by; delay of transfer of centre-of-pressure to the forefoot; delay of heel-rise and reduced peak vertical force; and net ankle plantar flexor muscle moment in terminal stance [11]. These compensations were obvious and marked in some cases but not others and overall no differences were detected other than for a delayed heel-rise in the early cases. Further work is required to determine more fully which of these metrics are the most sensitive to detect early changes. A second off-loading strategy for painful forefoot is to move the foot into a varus posture, especially if the medial MTP joints are involved, and this was detected in 2 of our cases. Fixed varus deformities of the rearfoot are reportedly present in about 2% of RA patients [24]. In our experience these are difficult to manage clinically. The long term functional consequences of this impairment are unclear.

Inflammatory synovitis and dysfunction of the peritalar joints and the tibialis posterior muscle-tendon unit are postulated mechanisms leading to instability of the subtalar and mid-tarsal joints [25]. This is clinically recognisable as pes planovalgus which has a reported prevalence of between 46–64% in RA [8,12,26,27]. The natural history of this progressive deformity is poorly understood but data from the present study suggests it may occur early and progress rapidly. In this study, clinical examination was used to successfully identify synovitis in and around the peri-talar joints. The consequence for these patients may be the progressive development of pes planovalgus. Indeed, 9/12 patients on standing had an exaggerated valgus heel posture above the normal values. Furthermore, we have previously described collapse of the medial longitudinal arch accompanied by an increase in the maximum rearfoot eversion reached during stance when walking [8,12,13]. These two motion deficits were clearly identified in this patient cohort, albeit two cases with moderately severe varus heel deformity appreciably skew the data. Finally, collapse of the medial longitudinal arch creates a larger midfoot weightbearing surface and in the early RA cases this was 21% greater than healthy controls.

In the planovalgus foot, the gastrocnemius-solues complex shows evidence of increased activity in an attempt to minimise the valgus deformity [28]. The third rocker function of the foot occurs about the forefoot as the heel rises in late stance and this is controlled by concentric contraction of the ankle plantarflexors. However, under manual muscle strength tests, the gastocnemius-soleus muscle group is usually weak in RA patients. The associated functional consequence may be reduced ankle joint power during terminal stance and this was detected for the RA patients in this study [11,28]. Since the net muscular moment was normal, the reduced ankle power must be caused by a reduction in the angular velocity at the joint. Factors contributing to reduce ankle joint angular velocity such as walking speed and reduced range of motion were detected amongst the patients but other gait-limiting factors and adaptations to impairments such as pain and deformity could not be fully accounted for. Finally, the blunting of the first peak of the vertical ground reaction force, suggests that some patients may have been cautious in loading the foot during initial foot contact phase, perhaps in an effort to lessen painful symptoms in the rearfoot. These functional changes in the rearfoot in early disease have important implications since we were able to successfully control motion and lessen symptoms by treating patients with customised foot orthoses [29,30]. However, the patients in the study by Woodburn *et al*. 2002 had median disease duration of 3 years suggesting we should attempt to identify rearfoot dysfunction even earlier and treat accordingly.

One of the limitations of this study is subject selection bias as these 12 cases were recruited following podiatry referral and may therefore represent only a subset of early RA patients with severe foot involvement. The introduction of Early Arthritis Clinics should permit future access to a more generalisable population. The sample size was also too small to permit formal hypothesis testing or statistical analysis of association between impairment and biomechanical data. Finally, due to the current limitations of gait analysis, our biomechanical foot models are insensitive to detection of functional changes at small but important single joints such as the subtalar, talonavicular, and lesser toe MTP joints which are involved in the disease process.

## Conclusion

In conclusion, in this cohort of RA patients with early disease we were able to detect moderate to high levels of foot impairment and associated disability. Furthermore, gait analysis detected subtle but functionally important changes to the biomechanical function of the foot. These findings need to be confirmed in a larger population with a fuller exploration of the relationship between disease activity, impairment and function in the foot. This information may be useful to distinguish those patients with a poor prognosis since a therapeutic window of opportunity may also exist for adjunct physical interventions such as corrective orthoses.

## Competing interests

The author(s) declare that they have no competing interests.

## Authors' contributions

**DET **conceived of the study, participated in the design of the study and carried out the gait analyses.

**PSH **participated in the design of the study and the coordination of the gait analyses.

**PE **participated in the design of the study and its coordination.

**JW **conceived of the study, participated in the design of the study, and conducted the gait data analysis and preparation.

All authors read and approved the final manuscript.

## Pre-publication history

The pre-publication history for this paper can be accessed here:



## Figures and Tables

**Table 1:** Demographic, clinical and foot impairment/disability scores for 12 RA patients and normal reference values for 12 age and sex matched able-bodied adults

**Patient/Age/Foot**	**DMARD**	**BMI (kg/m^2^)**	**DAS28 (0–10)**	**Erosive Disease**	**LFIS_IF _(0–21)**	**LFIS_AP _(0–30)**	**SJC (0–14)**	**TJC (0–14)**	**PJC (0–14)**	**SI_FF _(0–12)**	**SI_RF _(0–7)**	**RSFP (deg)**
1/43/L	MTX	30.5	7.3	-	14	19	14	14	4	1	0	-5 valgus
2/48/R	-	26.3	6.2	-	11	12	11	5	3	0	0	6 varus
3/36/R	SSZ	19.5	5.5	-	17	23	8	12	5	3	0	-4 valgus
4/55/L	MTX	27.4	5.0	+	6	8	0	2	0	2	1	-9 valgus
5/50/R	-	27.0	4.1	-	11	14	9	9	2	0	0	-4 valgus
6/27/R	MTX	25.1	4.1	Missing	9	10	1	9	2	0	1	-8 valgus
7/44/L	MTX, SSZ, HCQ	35.1	3.7	-	11	6	0	1	0	5	0	-2 valgus
8/32/R	TNFα, MTX	21.9	3.4	-	12	22	0	7	4	9	2	-7 valgus
9/58/R	MTX	25.0	2.7	-	10	3	1	10	1	0	0	-6 valgus
10/60/L	MTX	38.9	2.5	+	10	15	0	1	0	6	2	7 varus
11/43/R	SSZ	28.5	2.3	+	7	5	0	3	1	10	1	-8 valgus
12/63/L	MTX, HCQ	32.0	2.3	-	10	7	0	0	0	2	2	-9 valgus

Median 46	-	27.2	3.9		11	11	1	6	2	2	1	-6 valgus
Range 27–63	-	19.5–35.1	2.3–7.3		6–17	3–23	0–14	0–14	0–5	0–10	0–2	-9 valgus-7 varus
**Reference values **(n = 12)												
Median 47	-	23.7	-	-	1	0	0	0	0	1	0	-3 valgus
Range 27–64	-	19.1–31.8	-	-	0–4	0–2	0–2	0–1	0	0–2	0–2	-8 valgus-1 varus

DMARD = disease-modifying antirheumatic drug; MTX = methotrexate; SSZ = sulphasalazine; HCQ = hydroxychloroquine; TNFα = tumour necrosing factor α blockade; BMI = body mass index; DAS28 = disease activity score using 28 joint counts; LFIS_IF _= Leeds Foot Impact Scale-Impairment/Footwear subscale score 9 range 0–21); LFIS_AP _= Leeds Foot Impact Scale-Activity Limitation/Participation Restriction subscale score (range 0–30); SJC = swollen joint count (range 0–14); TJC = tender joint count (range 0–14); PJC = painful joint count (range 0–14); SI_FF _= structural index for forefoot; SI_RF _= structural index for rearfoot. RSFP = relaxed standing foot posture.

**Table 2:** Gait characteristics in patients with early rheumatoid arthritis and age and sex matched able-bodied adults

**Parameter**	**Variable**	**Able-bodied Adults (n = 12)**	**Early RA Patients (n = 12)**	**Mean Difference**	**95%CI Difference**
**Spatiotemporal**	Walking speed (m/s)	1.30 (0.17)	1.05 (0.20)	-0.24	(-0.41, -0.08)
	Double-support (%GC)	15.8 (3.4)	19.3 (4.7)	3.5	(0, 7.0)
**Kinematics**	Initial foot contact angle (deg)	13.8 (2.4)	14.2 (4.5)	-0.4	(-3.5, 2.7)
	Terminal stance heel rise (deg)	-85.7 (5.4)	-78.9 (9.6)	6.7	(0.1, 13.5)
	Minimum arch height (mm)	31 (5)	29 (7)	-2	(-8, 3)
	Peak eversion (deg)	-4.4 (3.4)	-5.5 (9.8)	-1.1	(-7.5, 5.4)
**Kinetics/Moments/Power**	Peak force loading response (BW)	1.11 (0.12)	1.05 (0.12)	-0.07	(-0.17, 0.04)
	Peak force terminal stance (BW)	1.12 (0.62)	1.12 (0.61)	0	(-0.05, 0.05)
	Peak plantarflexion moment (Nm/kg)	-1.6 (0.1)	-1.5 (0.1)	0	(-0.1, 0.1)
	Peak ankle joint power (W/kg)	4.6 (1.6)	3.4 (1.0)	-1.3	(-2.4, 0.2)
**Foot pressure**	CoP at 50% foot length (% stance)	44.2 (5.0)	44.3 (10.8)	0.1	(-7.2, 7.4)
	Lesser toes contact area (cm^-2^)	8.1 (1.9)	7.6 (3.1)	-0.5	(-2.7, 1.7)
	Midfoot contact area (cm^-2^)	19.4 (6.5)	24.6 (7.2)	5.3	(-0.6, 11.1)
	Forefoot peak pressure (kPa)	553 (193)	672 (255)	119	(-73, 311)

CI = confidence interval; BW = body weight (vertical ground reaction force normalised to body weight)
